# Correlation of Michigan neuropathy screening instrument, United Kingdom screening test and electrodiagnosis for early detection of diabetic peripheral neuropathy

**DOI:** 10.1186/s40200-016-0229-7

**Published:** 2016-03-25

**Authors:** Hamid R. Fateh, Seyed Pezhman Madani, Ramin Heshmat, Bagher Larijani

**Affiliations:** 1 Shariati Hospital, Tehran University of Medical Sciences (TUMS), Tehran, Iran; 2 Hazrat Fateme Reconstruction Surgery Hospital, Physical Medicine and Rehabilitation Department, Iran University of Medical Sciences (IUMS), Tehran, Iran; 3 Chronic Diseases Research Center, Endocrinology and Metabolism Population Sciences Institute, Tehran University of Medical Sciences (TUMS), Tehran, Iran; 4 Endocrinology and Metabolism Research Center, Endocrinology and Metabolism Clinical Sciences Institute, Tehran University of Medical Sciences (TUMS), Tehran, Iran

**Keywords:** Diabetic peripheral neuropathy, Electrodiagnosis, Michigan neuropathy screening instrument, United Kingdom screening test

## Abstract

**Background:**

Almost half of Diabetic Peripheral Neuropathies (DPNs) are symptom-free. Methods including questionnaires and electrodiagnosis (EDx) can be fruitful for easy reach to early diagnosis, correct treatments of diabetic neuropathy, and so decline of complications for instance diabetic foot ulcer and prevention of high costs. The goal of our study was to compare effectiveness of the Michigan neuropathy screening instrument (MNSI), United Kingdom screening test (UKST) and electrophysiological evaluation in confirming diabetic peripheral neuropathy.

**Methods:**

One hundred twenty five known diabetes mellitus male and female subjects older than 18 with or without symptoms of neuropathy comprised in this research. All of them were interviewed in terms of demographic data, lipid profile, HbA1C, duration of disease, and history of retinopathy, so examined by Michigan neuropathy screening instrument (MNSI), United Kingdom screening test (UKST), and nerve conduction studies (NCS). The collected data were analyzed by SPSS software 18.

**Results:**

One hundred twenty five diabetic patients (70 female, 55 male) were recruited in this study with a mean age of 58.7 ± 10.2, and mean duration of diabetes was 10.17 ± 6.9 years. The mean neuropathy score of MNSI and UKST were 2.3 (1.7) and 4.16 (2.9), respectively. Each instrument detected the peripheral neuropathy in 78 (69 %) and 91 (73 %) of patients, respectively. There was a significant relationship between number of neuropathies and mean of diabetes duration and development of retinopathy in both questionnaire evaluations and NCS. By nerve conduction study, neuropathy was detected in 121 (97 %) diabetic patients were reported in order 15 (12 %) mononeuropathy (as 33 % sensory and 67 % motor neuropathy) and 106 (85 %) polyneuropathy (as 31 % motor and 69 % sensorimotor neuropathy).

**Conclusions:**

As regards NCS is an objective, simple, and non-invasive tool and also can determine level of damage and regeneration in peripheral nerves, this study suggests electrodiagnosis as a convenient option for screening, confirming, and follow up of diabetic peripheral neuropathy.

## Background

The most common complication of diabetes mellitus is neuropathy as a consequence of chronic hyperglycemia. Advanced glycation products, aldose reductase, protein kinase C, polyol and some cytokines are among neurotoxic byproducts involved in pathophysiology of hyperglycemia induced diabetic peripheral neuropathy [1–7].

There is a wide spectrum of nerve involvement in diabetes with wide variety of manifestation from focal to diffuse, symmetric or asymmetric and autonomic symptoms [8].

Diabetic peripheral neuropathy (DPN) is the most common acquired neuropathy and one of the main complications of diabetes mellitus (DM) due to high rate of its prevalence, hospitalizations, morbidities and mortalities.

The valid prevalence is unknown through various criteria and methods for characterizing neuropathy, however, based on prior studies, the prevalence of DPN approximated 10–90 % generally and 16–87 % in Iran [9, 10].

DPN is diagnosed by symptoms and signs, but given that almost half of DPNs are symptom-free, other methods including questionnaires and electrodiagnosis (EDx) can be fruitful for easy reach to early diagnosis, correct treatments of diabetic neuropathy, and so decline of complications for instance diabetic foot ulcer and prevention of high costs [11–14].

Michigan neuropathy screening instrument (MNSI) and United Kingdom screening test (UKST) with questions about location and severity of clinical signs and symptoms of neuropathy are commonly used to assess DPN [13]. based on physician’s clinical examination and patient’s self-report.

The goal of our study was to compare effectiveness of the Michigan neuropathy screening instrument (MNSI), United Kingdom screening test (UKST) and electrophysiological evaluation in confirming diabetic peripheral neuropathy.

## Methods

This cross sectional study was performed in the Endocrinology and Metabolism Research Institute of Tehran University of Medical Sciences. One hundred twenty five known diabetes mellitus subjects confirmed by endocrinologists with more than 18 years of age, both male and female with or without symptoms of neuropathy comprised in this research.

Patient with psychological problem, potential for peripheral neuropathy consisting of hereditary sensory neuropathy, vitamin B12 or folate deficiency, paraneoplastic diseases, autoimmune conditions, organs failure, hypothyroidism prolonged phenytoin or immunosuppressive drugs consumption, and ethanol abuse were excluded from the study [13].

All of them were interviewed in terms of demographic data, lipid profile, HbA1C, duration of disease, and history of retinopathy, so examined by Michigan neuropathy screening instrument (MNSI), United Kingdom screening test (UKST), and nerve conduction studies (NCS).

### Instrument

#### Michigan neuropathy screening instrument (MNSI)

MNSI has two steps to assess history of neuropathic symptoms and physical examination to evaluate the appearance and sensation of feet. An objective test with four questions included foot skin inspection for deformities, dry skin, calluses, infections, fissures, and ulcer, ankle reflex, and vibration sensation tested by a 128 HZ tuning fork placed over great toe. The test was completed by expert physician. A score ≥ 2 is considered abnormal. Abnormality in each item gets grades 0.5 to 1 and at lease more than 2 abnormal items needed to reach the score of neuropathy [15].

#### United Kingdom screening test (UKST)

A simple, subjective and symptom-based instrument which is composed of five questions about type, severity and location of symptoms and maximum 9 scores. Its cut-off point is ≥ 2 [16, 17].

#### Electrophysiological assessment

NCS is the most fruitful component of the electrodiagnostic evaluation, so a simple, noninvasive, objective, and sensitive measurement which is intended as a gold standard test for corroborating the diagnosis of peripheral neuropathy [18–22].

There is no general consensus for polyneuropathy criteria in NCS in the face of multiple performed investigations [18]. Because of DPN is length-dependent neuropathy, lower extremity nerves are probably involved more, we designed our study accordingly [23].

NCS consists of bilateral peroneal and tibial nerves compound muscle action potential (CMAP) and sural nerves sensory nerve action potential (SNAP), nerve conduction velocity (NCV), amplitude, and distal latency performed by board-certified physiatrists and electromyographers via using 2-channel Oxford (Medelec-Synergy) electromyography instrument. Normal values were considered based on valid data [24].

For peroneal nerve CMAP, recording electrode was attached on extensor digitorum brevis and peroneal nerve was stimulated distally at ankle, lateral to tibialis anterior tendon and proximally a few centimeters distal to the fibular head.

For tibial nerve CMAP, recording electrode was attached on abductor hallucis muscle and tibial nerve was stimulated in the popliteal fossa and proximally posterior to the medial malleolus proximal to the flexor retinaculum.

For sural nerve SNAP the recording electrode was attached on posterior aspect of lateral malleolus and the stimulator was positioned 14 cm proximally in the posterior aspect of leg’s midline [25].

### Ethical considerations

The Research Committee of Endocrinology and Metabolism Research Institute of Tehran University of Medical Sciences approved the study. Ethically each patient signed the informed consent form before participation in the study.

### Statistical analysis

To explain continuous and qualitative variables, mean [standard deviation (SD)] and frequency (percentage) were utilized, respectively. Continuous variables were compared by independent sample *T*-test, or Mann-Whitney U test whenever the data did not appear to have normal distribution or when the assumption of equal variances was violated across the study groups. Pearson chi-square test was used to evaluate the proportion of the qualitative variables between groups. In this study, a *P* value <0.05 as statistically significant, in addition, SPSS software version 18.0 was applied.

## Results

One hundred twenty five diabetic patients were recruited in this study. They included 70 (56 %) female and 55 (44 %) male with a mean age of 58.7 ± 10.2, ranging from 22 to 77 years of age, 17 (14 %) type 1 DM and 108 (86 %) type 2 DM. Mean duration of diabetes history was 10.17 ± 6.9 years. Demographic characteristics of the patients are reported in Table 1.

**Table 1:** Baseline and general characteristics of recruited patients

Variable	Mean ± SD
Female/Male	70/55
Age	58.7 (10.2)
DM1/DM2	17/108
Duration of disease	10.17 (6.9)
BMI	27.8 (4.3)
Height	161.3 (9.8)
Weight	72.3 (11.0)
HbA1C	7.9 (2.4)
Total cholesterol	203.8 (34.4)
LDL	117.4 (29.5)
HDL	42.6 (11.4)
Triglyceride	214.9 (98.8)

In the neuropathy evaluation, the mean score of MNSI and UKST were 2.3 (1.7) and 4.16 (2.9), respectively. In accordance with specified cut-off point in MNSI and UKST, peripheral neuropathy was detected in 78 (69 %) and 91 (73 %) of patients, respectively.

Classification of neuropathy severity based on UKST score as follows,

Healthy or without neuropathy (0–1): 34 (27 %), mild (2–4): 24 (19 %), moderate (5–6): 34 (27 %) and severe (7–9): 33 (26 %).

As reported in Table 2, there was a significant relationship between number of neuropathies and mean of diabetes duration in both questionnaire evaluations and NCS, however, according to HbA1C, not statistically significant difference in MNSI.

**Table 2:** Relationship between neuropathy and diabetes duration or HbA1C

Neuropathy	Yes	No	*P* value
DM duration (MNSI)	11.5 ± 6.4	7.8 ± 7.2	0.003
HbA1C (MNSI)	7.9 ± 2.2	7.8 ± 2.7	0.808
DM duration (UKST)	11.2 ± 7.09	7.15 ± 5.8	0.003
HbA1C (UKST)	8.2 ± 2.3	6.8 ± 2.3	0.024

Our results showed that hyperlipidemia (total cholesterol > 200 or triglyceride > 150) and obesity, body mass index (BMI) > 30 did not have significant difference with neuropathy in both questionnaire assessments and NCS.

Twenty six subjects of retinopathy (based on positive history of laser therapy) evaluated by MNSI, UKST and NCS, their *P*-values were statistically significant (0.050 and 0.025 respectively).

Neuropathy was diagnosed based on one or more abnormal findings in nerve conduction study, including distal latency, amplitude and velocity of conduction in the tested nerve and therefore 121 (97 %) neuropathic patients were reported in order 15 (12 %) mononeuropathy (as 33 % sensory and 67 % motor neuropathy) and 106 (85 %) polyneuropathy (as 31 % motor and 69 % sensorimotor neuropathy). Frequency of neuropathy based on involved nerves and assessment tests showed in Tables 3 and 4, respectively.

**Table 3:** Frequency of neuropathy based on involved nerve

Nerve	Amplitude	Latency	NCV	%
Right tibial	43.2 %	3.2 %	26.4 %	49.6 %
Left tibial	48.8 %	4.8 %	29.6 %	53.6 %
Right peroneal	68 %	35.2 %	40 %	75.2 %
Left peroneal	75.2 %	29.6 %	52 %	81.6 %
Right sural	49.6 %	17.6 %	25.6 %	52 %
Left sural	51.2 %	21.6 %	24 %	81.6 %

**Table 4:** Frequency of neuropathy based on assessment tests

Test	Type 1 DM	Type 2 DM
MNSI	16.9 %	83.1 %
UKST	15.6 %	84.4 %
EDx	14.2 %	85.8 %

In this study there is statistically significant agreement between electrophysiological assessment with UKST and MNSI in diagnosis of sensory neuropathy (*p* < 0.001). There is a strong correlation between the MNSI and UK total score (Pearson’s correlation coefficient = 0.43, *p* < 0.001).

By considering NCS as a comparative standard in this population of diabetic patients, the sensitivity of UKST was 63.93 %, the specificity 50 % and the positive/negative likelihood ratios were 1.28 and 0.72. For MNSI these were 75.21 %, 33.3 %, 1.13 and 0.74 respectively.

## Discussion

Diabetic peripheral neuropathy is a prevalent problem with secondary complications can cause different sufferings such limb amputation, also may expand before diagnosis and being symptomatic, following from this; the diagnosis of neuropathies is very important in suitable time in result. Owing to absence of a thorough diagnostic criteria and accurate instrument, various methods were designed for this purpose [11, 12, 26].

Diagnosis of neuropathy founded on physical examinations, questionnaires, nerve conduction studies and skin biopsies, however, initially researchers propounded some questionnaires in addition to physical examinations, but the results showed they are less efficient because of being essentially subjective, time consuming and its dependence on both patient and examiner, also unable to detect all cases of neuropathy and their severities [26].

In a study [27], slowed NCV was revealed in 100 % of lower and 48 % of upper extremities of tested diabetic patients. In another study, asymmetric distal neuropathy totally occurred in 84 % [28], and in some other studies [29, 30] also showed reduced sensory and motor NCVs in almost all participants.

Our research showed that percentage of discovered neuropathies by mentioned questionnaires are between 69 and 73 %; however, this amount for electrophysiologic assessment is between 85 and 97 % according to the selected criteria.

Our findings, as previous studies, showed that electrophysiological assessment in diabetes mellitus achieved higher sensitivity to diagnose motor and sensory neuropathies in comparison with screening questionnaires [31, 32], which explainable by higher sensitivity of electrophysiological studies in early detection of subclinical neuropathies.

According to the Table 4, the most of diagnosed neuropathies with MNSI, UKST, and NCS are related to type 2 diabetes mellitus and the reason of high prevalence of DPN in adult-onset diabetes can be further damage of large fiber nerves and more ability of these three tools for large fiber neuropathy screening [24, 33, 34].

## Conclusions

MNSI and UKST are sensitive screening instruments for routine evaluation of diabetic patients. As regards NCS is an objective, simple, and non-invasive tool and also can determine level of damage and regeneration in peripheral nerves, this study suggests electrodiagnosis as a convenient option for screening, confirming, and follow up of diabetic peripheral neuropathy. In addition, the role of NCS in early detection of subclinical neuropathies makes it a suitable test for periodic evaluation of diabetic patients even with normal MNSI and UKST screening studies.

## Abbreviations

BMI: body mass index
CMAP: compound muscle action potential
DM: diabetes mellitus
DPN: diabetic peripheral neuropathy
EDx: electrodiagnosis
IUMS: Iran University of Medical Sciences
MNSI: Michigan neuropathy screening instrument
NCS: nerve conduction study
NCV: nerve conduction velocity
SD: standard deviation
SNAP: sensory nerve action potential
TUMS: Tehran University of Medical Sciences
UKST: United Kingdom screening test
